# O3.1. NEUROTYPING UNTREATED FIRST EPISODE SCHIZOPHRENIA ON THE BASIS OF SLOW-WAVE RESTING-STATE DYNAMICS

**DOI:** 10.1093/schbul/sby015.199

**Published:** 2018-04-01

**Authors:** Tushar Das, Mingli Li, Lena Palaniyappan, Tao Li

**Affiliations:** 1 University of Western Ontario; 2 Mental Health Center, West China Hospital of Sichuan University

## Abstract

**Background:**

Coherence in the phase of oscillatory neuronal activity indicates functional interaction among brain regions at rest. Infra-slow fluctuations in BOLD signal (band 5 - 0.01 to 0.027 Hz - and band 4 - 0.027 to 0.073 Hz) has been observed using resting state fMRI when employing frequency-domain analysis, and has been previously shown to be altered in schizophrenia. In the current study, we examined the strength and the dynamic variance of phase coherence in these 2 bands (using a sliding window approach) among 6 large-scale brain networks (default-mode, fronto-parietal, salience, sensorimotor, visual, cerebellar) in 129 drug-naïve patients with schizophrenia and 197 healthy controls. Our motivation was to exploit the large-scale resting-state slow-wave oscillatory dynamics to parse the heterogeneity of schizophrenia.

**Methods:**

Four 6*6 matrices depicting patient vs. control differences in dynamic variance and mean of phase coherence (vPC and mPC respectively) in the slow 4 and slow 5 bands were constructed from resting state fMRI time series obtained from 6 networks based on 160 nodes of Dosenbach’s atlas. Deviations in mean/variance of phase coherence among the 6 networks were identified after FDR correction for each matrix (p<0.05). A latent profile analysis (LPA) was undertaken on the basis of the identified deviant features in the patient group. LPA is a Finite Mixture Modelling approach to identify naturally occurring sub-groups of patients on the basis of multivariable data. The identified subgroups were compared in terms of the severity of clinical symptoms across van der Gaag’s 5 factors of PANSS scale.

**Results:**

Patients with schizophrenia showed increased vPC between salience-sensorimotor and visual-cerebellar networks in band 5; decreased vPC between DMN-sensorimotor and DMN-cerebellar networks in band 4. Patients also had a decrease in mPC between DMN-visual and DMN-cerebellar networks in band 5. We were able to identify 3 subgroups of patients using LPA. SZ1 (n=28) and SZ2 (n=45) had higher overall burden of symptoms compared to SZ3 (n=56). SZ2 had the highest burden of negative syndrome score and showed most deviance from healthy controls (5 out of 7 features significantly different from the healthy cohort). SZ1 had the highest burden of positive syndrome scores and had 4 out of 7 features deviant from HC. In contrast, SZ3 had least deviation from HC (3 out of 7 features) and also had less symptom burden across all symptom dimensions.

**Discussion:**

Various abnormalities have been reported in the interactions among the large-scale networks in schizophrenia, with lack of consistency ascribed to syndromic heterogeneity. We illustrate how deviations in time-varying nature of slow-wave oscillations in resting state fMRI can be exploited to meaningfully reduce heterogeneity of this illness. The 3 subgroups thus identified not only show differential symptom burden but also exhibit hierarchical deviation from a normative group of healthy controls (SZ2>SZ1>SZ3>HC). To our knowledge this is the first attempt to stratify ‘neurotypes’ among drug-naïve patients with schizophrenia on the basis of large-scale network dynamics. Given the widespread availability of resting-fMRI data, we anticipate independent replication of our results in the near future.

